# The Potential of Food Fortification to Add Micronutrients in Young Children and Women of Reproductive Age – Findings from a Cross-Sectional Survey in Abidjan, Côte d’Ivoire

**DOI:** 10.1371/journal.pone.0158552

**Published:** 2016-07-06

**Authors:** Fabian Rohner, Magali Leyvraz, Amoin G. Konan, Lasme J. C. E. Esso, James P. Wirth, Augusto Norte, Adiko F. Adiko, Bassirou Bonfoh, Grant J. Aaron

**Affiliations:** 1 GroundWork, Flaesch, Switzerland; 2 Global Alliance for Improved Nutrition, Geneva, Switzerland; 3 Centre Suisse de Recherches Scientifiques en Côte d’Ivoire, Abidjan, Côte d’Ivoire; 4 Université Félix Houphouët-Boigny, Abidjan, Côte d’Ivoire; 5 Swiss Tropical and Public Health Institute, University of Basel, Basel, Switzerland; RTI International, UNITED STATES

## Abstract

Poor micronutrient intakes are a major contributing factor to the high burden of micronutrient deficiencies in Côte d’Ivoire. Large-scale food fortification is considered a cost-effective approach to deliver micronutrients, and fortification of salt (iodine), wheat flour (iron and folic acid), and vegetable oil (vitamin A) is mandatory in Côte d’Ivoire. A cross-sectional survey on households with at least one child 6–23 months was conducted to update coverage figures with adequately fortified food vehicles in Abidjan, the capital of and largest urban community in Côte d’Ivoire, and to evaluate whether additional iron and vitamin A intake is sufficient to bear the potential to reduce micronutrient malnutrition. Information on demographics and food consumption was collected, along with samples of salt and oil. Wheat flour was sampled from bakeries and retailers residing in the selected clusters. In Abidjan, 86% and 97% of salt and vegetable oil samples, respectively, were adequately fortified, while only 32% of wheat flour samples were adequately fortified, but all samples contained some added iron. There were no major differences in additional vitamin A and iron intake between poor and non-poor households. For vitamin A in oil, the additional percentage of the recommended nutrient intake was 27% and 40% for children 6–23 months and women of reproductive age, respectively, while for iron from wheat flour, only 13% and 19% could be covered. Compared to previous estimates, coverage has remained stable for salt and wheat flour, but improved for vegetable oil. Fortification of vegetable oil clearly provides a meaningful additional amount of vitamin A. This is not currently the case for iron, due to the low fortification levels. Iron levels in wheat flour should be increased and monitored, and additional vehicles should be explored to add iron to the Ivorian diet.

## Introduction

The burden of malnutrition in Côte d’Ivoire is among the highest in the world. According to recent estimates, almost one-third of preschool-aged children (PSC) are stunted, and suboptimal intrauterine growth, as expressed for small-for-gestational age newborns, affects over a quarter of newborns [1, 2]; anemia affects three-quarters of Ivorian PSC and more than half of women of reproductive age (WRA) [2].

At the national level, vitamin A deficiency (VAD) in WRA (0.8%) was surprisingly low, as was iron deficiency prevalence (defined as low serum ferritin concentrations) among PSC (16%) and WRA (17%) in 2007. In contrast, VAD was high among PSC (24%). For Abidjan, the data showed a slightly better situation, except for iron deficiency in PSC, which was close to 26% [3].

Interventions addressing undernutrition, in particular among PSC, have repeatedly scored high in the Copenhagen consensus for cost-effective strategies to address the world’s biggest challenges [4]. Large-scale food fortification is considered a highly cost-effective approach to deliver micronutrients, in particular if a wide range of the population is in need of additional micronutrient intakes [5]. Further, for some micronutrients with a very narrow window of opportunity, it is difficult to reach the target population through a targeted approach, e.g., folic acid effectively reduces the risk of neural tube defects if provided to future pregnant women prior to conception, but not when given later on during pregnancy [6].

In Côte d’Ivoire, the Ministry of Health and the Fight against AIDS (Ministère de la Santé et de la Lutte contre le Sida) adopted multiple strategies to combat malnutrition, micronutrient deficiencies, and anemia, including iron and vitamin A supplementation, dietary counseling to encourage food diversification, food fortification, and public health measures to aid in the reduction of malaria and other parasitic diseases. Further, pregnant women are provided with iron and folate tablets during prenatal and postpartum consultations [7]. Efforts to reduce VADs in Côte d’Ivoire include the administration of vitamin A capsules to PSC aged 6–59 months, in conjunction with routine immunization programs, e.g., the Expanded Programme on Immunization (EPI)-Plus [8]. To endorse the government’s efforts, several national and international stakeholders supported a fortified food program (Programme Ivoirien de la Promotion des Aliments Fortifiés [PIPAF]) to fortify wheat flour with electrolytic iron and folic acid, and refined vegetable oil with vitamin A, starting in 2007.

In the context of the PIPAF on the promotion of fortified foods, and following the laws pertaining to the fortification of vegetable oils and wheat flour in 2007, the production of vegetable oils fortified with vitamin A was estimated to have risen from 55% in 2005 to 80% in February 2008. However, data from a national coverage survey in late 2009 found after quantitative analysis of vitamin A content in 2,350 oil samples that only about a quarter were adequately fortified (mandated level of 8 μg/g ± 20%), with only 20% coverage in Abidjan [9]. Fortification of wheat flour with iron and folic acid was nonexistent in 2005, but during 2008, all wheat mills commenced flour fortification. The same survey found that coverage with flour fortified close to levels previously mandated (60 ± 12 parts per million [ppm] as electrolytic iron) was about 50%, while for Abidjan the coverage figure was slightly lower at 43%.

This coverage survey was piggybacked onto a survey focusing on infant and young child nutrition, as it presented a unique opportunity to update the picture of coverage after interruptions of the regulatory and health system sector due to a profound post-electoral crisis in 2010, in order to provide guidance to national policy makers but also to illustrate the importance of continued policy support in fortification programs.

## Materials and Methods

### Study design and setting

A cross-sectional, three-stage cluster household survey was conducted in Abidjan in September 2014. In the first stage of sampling, nine primary sampling units (PSUs), which consisted of the smallest census unit and contained about 200 households, were randomly selected in each of the 10 communes in Abidjan, with the probability of selection for each PSU being proportional to the number of households in that PSU. Because at the time of the survey the data from the 2014 national census were not available at the level of the PSU and because the identification of households with at least one child aged under 2 years old was required, the data collection teams conducted a mini-census to identify households with a child under 2 years of age and to update the estimated PSU size. Thereafter, a random selection of 13 households with at least one child aged 0–23 months was conducted in each selected PSU. If several eligible children were living in a selected household, only one child was randomly selected using a Kish table [10].

### Ethics and consent

Approval for the study was granted by the Ethics Committee of the Ministry of Health in Côte d’Ivoire (Comité National d’Ethique et de la Recherche, clearance number 55/MSLS/CNR-dkn). Inclusion in the survey was dependent on the household head being willing to participate in the survey and the caregiver giving written informed consent for herself and the child. As part of the survey, mid-upper arm circumference was measured and any caregiver or child diagnosed with severe acute malnutrition was referred to the nearest health facility. All participating households were given two bars of soap and an information brochure for adequate infant and young child feeding practices to thank them for the time taken for the interview.

### Enrollment of participants

After the household selection and consent procedure to take part in the survey, a household roster to collect data on all household members (sex, age, education) was completed. Subsequently, one child aged 6–23 months was randomly selected along with his/her caregiver. “Caregiver” was defined as being the person with the greatest responsibility for taking care of the child, which, in the majority of the cases, was the biological mother, but was in some cases someone else (e.g., if the mother was absent for an extended period of time, had passed away, or was working outside the home). The caregiver then answered questions on demographics, health, and socioeconomic status (housing quality and assets, water/hygiene and sanitation, electricity), as well as on frequency and quantity of purchases of oil and wheat flour products and bouillon cubes. For flour consumption, because most households consume pre-processed wheat flour-based products, the mother-child pairs were administered a 7-day recall module for wheat flour products.

### Food sampling and analysis

During the interview, the respondents were asked whether they could provide a small amount of vegetable oil and salt and, if available, a sample of approximately 15 mL (oil) and/or approximately 10–20 g (salt) was collected. For flour, because only very few households have flour at home, flour samples were collected from up to three randomly selected shops and up to three bakeries (if available) in each PSU. These samples were mixed in equal proportions to make a composite sample for each PSU to estimate the fortification exposure intensity.

After collection at the households, shops, and/or bakeries, oil and flour samples were stored in boxes in the dark in air-conditioned rooms before being shipped to Germany for laboratory analysis. Laboratory analyses were conducted by BioAnalyt (Potsdam, Germany): iCheck Iron for iron in wheat flour, iCheck Chroma 3 for retinyl palmitate in vegetable oil, and iCheck Iodine for iodine in salt [11–14]. For wheat flour, because only a few samples for each cluster were collected and because the household could easily purchase flour-containing products outside the PSU they resided in, it was deemed appropriate to pool the results of all samples and to assign the mean iron concentration to each household. For vegetable oil, of all samples collected from the households within the same PSU, a composite sample was generated and analyzed, and the results of the composite sample were assigned to the households of the PSU.

To determine, whether measured fortificant levels fell into the category of adequacy according to national standards, the concentrations presented in Table 1 were used.

**Table 1:** Legally mandated fortification levels in Côte d’Ivoire for wheat flour, vegetable oil and salt.

Vehicle	Fortificant	Mandated levels at			
		Importation	Production	Sales points	Reference
Salt	Iodine (as KIO_3_)	80–100 mg/kg	50–80 mg/kg	30–50 mg/kg	[15]
Vegetable oil	Retinyl palmitate or equivalent	8 μg retinol equivalents/g			[16]
Wheat flour	Electrolytic iron	60 mg/kg			[17]
	Ferrous fumarate	30 mg/kg			[18]
	Folic acid	1.5 mg/kg			[17]

### Data management and statistical analysis

All field data were double entered and cross-checked using EpiData Entry (http://www.epidata.dk). Statistical analyses were conducted using SPSS (version 21, IBM Corporation, Armonk, NY, USA) and R (version 3.1.0). Continuous data were checked for skewness using the Cox test (coefficient of skewness divided by the standard error of skewness), as well as by examination of the frequency distribution. The relationship between two categorical variables was analyzed by the chi-square test and between continuous variables by independent-sample t-test or one-way analysis of variance (ANOVA). P-values of 0.05 were considered statistically significant throughout. Cluster and stratified sampling was accounted for when calculating measures of precision.

The calculation of the daily vitamin A intake from oil was done as follows:

1. The daily quantity of oil consumed at the household was calculated from the quantities purchased each time or money spent on purchasing oil and the reported frequency of purchase; the amount of oil in liters was then converted to oil in kg (using the conversion factor of 0.8875 g/ml at 25°C [19].
2. The amount of vitamin A consumed per household was obtained using the measured concentration of vitamin A from the oil specimen analyses in mg retinol equivalents (RE)/kg multiplied by the amount of oil (in kg) consumed on a daily basis.
3. The number of adult male equivalents (AMEs) was then calculated using established conversion algorithms [20]. For this, the number of household members in each age group was determined, and the number of AMEs summed together, enabling the calculatation of oil consumption per individual household member.

The percentage of the recommended nutrient intakes (RNIs) was obtained from the World Health Organization/Food and Agriculture Organization of the United Nations (WHO/FAO) [21] and combined with additional amounts of vitamin A coming from the vegetable oil. Results indicating a consumption of > 200 mL/day for one AME were excluded, as it was deemed that this was not physiologically very plausible. It is noteworthy that for the calculation of the mean intake, children and women from households reporting not having consumed vegetable oil or wheat flour were included as well, but they represented only a very small proportion and, thus, did not affect results.

The AME approach was also used to estimate the consumption of bouillon cubes; this condiment was included not because fortification is mandatory, but to assess the potential of this food vehicle for fortification in urban Côte d’Ivoire.

For flour, because only very few households use flour at home for daily preparation, a different approach was taken: The caregiver and the child were asked about the frequency and quantity of a range of flour-containing foods [22]. To do so, separately for the caregiver and the child, the frequency of eating a certain product was asked; for the estimation of quantity, a picture catalogue with portion sizes of the 20 most commonly consumed flour-containing products was used. After data entry, the responses were converted to total weights of that specific food and then, based on a range of recipes to create average flour content of the foods, into grams of flour consumed. To these intake data, the intake from wheat flour at the households was added (using the AME approach described above) for those households that reported purchasing flour. Mean iron content of the flour of the composite sample from a PSU was used to calculate the additional amount of iron in the flour; the comparison base of WHO/FAO [21] RNIs was used again. Reported intakes of > 600 g/day for WRA and 150 g/day for children aged 6–23 months were considered as outliers and removed from further analysis.

The multidimensional poverty index (MPI) was constructed according to Alkire and Santos [23], whereby living standards, education, health and nutrition, and household assets are combined to create an index ranging from 0 to 1, where 0 indicates no poverty and a MPI of ≥ 0.33 categorizes the household as being at risk for poverty.

## Results

In total, in 1,113 out of 1,170 households, an eligible mother-/caregiver-child pair was present at the time of the interview and the household consented to participate in the survey, yielding a response rate of 95.1%. Table 2 provides an overview of the household and demographic characteristics of the survey sample. A slightly larger household size than that reported by the most recent Demographic and Health Survey (DHS) of 5.3 members [2] was found in this survey (6.1 members), but this may be explained by the selection of households with at least one child under 2 years old, which leaves out households without children. In 95% of the selected households, only one child of that age resided there at the time of the survey.

**Table 2:** Household and Demographic Characteristics of the Survey Sample, Abidjan, 2014.

Variable	N	Mean/Percentage (95% CI)^a^
**Household level**		
Household size, n	1,113	6.10 (5.81, 6.38)
Household dependency ratio, (-)^b^	1,113	0.58 (0.54, 0.63)
MPI score ≥ 0.33, %^c^	1,106	21.0 (16.6, 26.3)
Electricity, %	1,106	97.0 (82.1, 99.6)
Clean cooking fuel, %	1,106	77.7 (70.9, 83.3)
Improved flooring, %	1,106	98.5 (95.7, 99.5)
Safe drinking water source, %	1,106	99.0 (98.0, 99.5)
Safe toilet sanitation, %	1,106	55.1 (46.6, 63.3)
Any household member 5–14 years NOT currently attending school, %	1,106	17.8 (15.0, 21.0)
**Caregiver **		
Age (years)	1,113	29.0 (28.4, 29.6)
≥ 5 years of education, %	1,113	49.5 (44.4, 54.6)
**Child **		
Age (months)	1,113	10.8 (10.3, 11.2)
Sex, female, %	1,113	46.3 (41.5, 51.2)

^a^ All values are mean, median, or percent as indicated, and are weighted to correct for unequal probability of selection. Mean was used as the measure of central tendency for normally distributed variables. Median was used for non-normally distributed variables.

^b^ Household dependency ratio = (Number of household members under 15 years old and over 64 years old)/(Number of household members between 15 and 64 years old)

^c^ MPI score ≥ 0.33 is considered at risk of acute poverty.

About a fifth of households were categorized as poor, yet the vast majority of the households had access to safe drinking water and had improved flooring materials. In contrast, only just over half of the households had access to safe toilet sanitation.

Almost all (98%) households reported purchasing palm oil and about half of the respondents gave a single brand as the purchased brand, with the other half not knowing the brand. Similarly, the vast majority (92%) of households had salt at home at the time of the survey, but almost no respondent could provide a brand name. This is likely due to the fact that over 80% of salt purchased was purchased in bulk. For wheat flour, only 12% of the households actually had flour at home; in contrast, most of the adult caregivers reported having consumed some flour containing product in the past 24 hrs (92%), while for children, the proportion is lower (52%).

Mean concentration of retinyl palmitate in vegetable oil was 9.6 μg RE/g (95% CI: 9.3, 9.6), without poor/non-poor differences; mean salt iodine content was 62.1 mg/kg (95% CI: 59.5, 64.6), with salt sampled from poorer households showing a slightly but statistically significantly higher mean iodine content (60.7 vs. 67.6; p = 0.043). Mean iron content of wheat flour was 25.0 mg/kg; calculation of 95% CI was not possible because, for data analysis, the mean iron content of all samples measured was assigned to each interviewed household.

**Fig 1:**
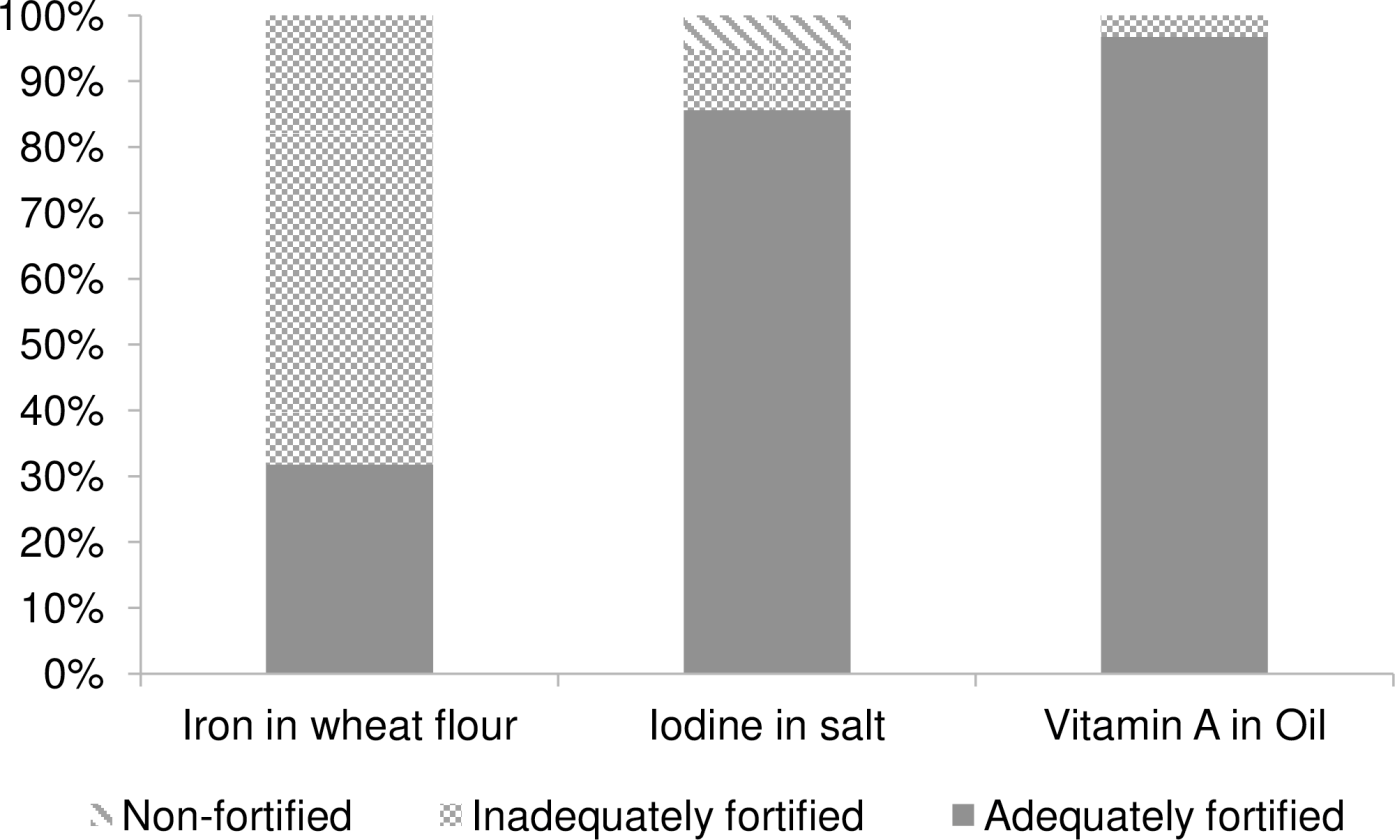
Household Coverage with Unfortified and Inadequately and Adequately Fortified Wheat Flour, Salt, and Vegetable Oil, Abidjan, 2014. “Adequately fortified” is defined according to the most recent national regulations [15, 16, 18]: > 30 mg iron/kg of flour as ferrous fumarate, > 30 mg iodine/kg salt as potassium iodate, and > 8 μg RE/g oil as retinyl palmitate.

Fig 1 provides an overview of household coverage of these vehicles. Results show that the majority of vegetable oil samples (97%) and of salt samples (86%) were adequately fortified; however, for wheat flour, only just over 30% had adequate levels of iron. Yet no flour sample analyzed was completely unfortified, indicating that efforts to fortify are being undertaken by the millers. For salt, while there is only a small proportion of non-iodized salt (5%), more than 9% is iodized to some extent, although not to adequate levels; the remainder is adequately iodized.

The mean daily intakes of vegetable oil and wheat flour for children aged 6–23 months and for WRA are shown in Table 3. For children, the oil and flour intakes are shown for different age categories, as the intake in this age range changes rapidly with increasing age. There were no significant differences in oil or flour intake between the women from poor and non-poor households. For children, the differences are significant for oil consumption in the 12–23 months age group and in the 9–11 months age group for wheat flour consumption, with those children from non-poor households consuming larger quantities. Also, although not surprising, children from the oldest age groups mostly consume larger amounts of oil and wheat flour than the younger age groups.

**Table 3:** Daily Oil and Wheat Flour Consumption Stratified by Population Group and MPI Score, Abidjan, 2014^a^.

Variable	N	Mean (95% CI)						P-value^d^
		All^b^		Poor^c^		Non-poor		
**Vegetable oil, mL/d**								
*Children*								
6–8 months	125	8.4	(7.0, 9.8)^a^	9.7	(5.0, 14.4)^a^^,^^b^	8.0	(6.6, 9.4)^a^	0.498
9–11 months	102	9.6	(6.6, 12.5)^a^	7.6	(6.0, 9.2)^a^	10.0	(6.5, 13.5)^a^	0.219
12–23 months	532	13.5	(11.9, 15.1)^b^	11.5	(9.2, 13.7)^b^	14.2	(12.5, 15.9)^b^	0.041
*Women of reproductive age*								
15–49 years	1,053	30.1	(27.1, 33.0)	27.3	(23.2, 31.4)	30.8	(27.4, 34.1)	0.163
**Wheat flour, g/d**								
*Children*								
6–8 months	130	11.3	(5.7, 16.8)^a^	6.9	(1.8, 12.0)^a^	12.3	(5.6, 19.1)^a^	0.183
9–11 months	104	19.7	(14.8, 25.4)^b^	12.0	(6.7, 17.4)^a^	21.4	(15.1, 27.8)^a^	0.035
12–23 months	536	33.0	(29.2, 36.8)^c^	30.9	(23.9, 37.9)^b^	33.3	(29.6, 37.0)^b^	0.478
*Women of reproductive age*								
15–49 years	1,068	125.4	(112.3, 138.4)	123.0	(106.0, 140.0)	126.0	(111.4, 140.6)	0.756

^a^ All values are mean and are weighted to correct for unequal probability of selection.

^b^ Means in a column without a common letter differ, P-value < 0.05.

^c^ MPI score ≥ 0.33 is considered at risk of acute poverty.

^d^ P-value calculated using the independent samples t-test, adjusted for unequal probability of selection.

Micronutrient contributions from the included food vehicles are shown in Table 4. Because the vitamin A contents in the different oil samples were relatively consistent and because for the iron content in wheat flour a mean concentration was assigned to all participating households, Tables 2 and 3 are not very different with regard to poor vs. non-poor or for the different age groups in children.

**Table 4:** Vitamin A and Iron Contribution from Fortified Vegetable Oil and Wheat Flour, Expressed as % of RNI, Stratified by Population Group and MPI Score, Abidjan, 2014^a^.

Variable	N	Percentage (95% CI)						P-value^c^
		All		Poor^b^		Non-poor		
**Vegetable oil, % RNI of vitamin A**								
*Children*								
6–8 months	125	18.3	(14.9, 21.9)^a^	22.8	(10.6, 35.2)^a^^,^^b^	17.1	(14.1, 20.0)^a^	0.375
9–11 months	102	21.0	(13.4, 28.5)^a^	15.9	(12.2, 19.6)^a^	22.2	(13.2, 31.3)^a^	0.198
12–23 months	532	28.9	(25.4, 32.5)^b^	24.7	(20.1, 29.4)^b^	30.4	(26.4, 34.4)^b^	0.045
*Women of reproductive age*								
15–49 years	1,042	37.4	(32.8, 42.0)	33.5	(28.2, 38.8)	38.4	(33.1, 43.7)	0.152
**Wheat flour, % RNI of iron**								
*Children*								
6–8 months	97	2.8	(0.9, 4.8)^a^	1.5	(0.0, 3.4)^a^	3.1	(0.9, 5.4)^a^	0.261
9–11 months	104	5.3	(3.8, 6.8)^a^	3.7	(1.9, 4.8)^a^	5.7	(4.0, 7.4)^a^	0.044
12–23 months	536	13.9	(12.3, 15.5)^b^	12.9	(9.9, 15.8)^b^	14.0	(12.5, 15.6)^b^	0.418
*Women of reproductive age*								
15–49 years	1,055	17.8	(15.7, 19.8)	18.0	(14.9, 21.2)	17.7	(15.5, 19.9)	0.830

^a^ All values are mean and are weighted to correct for unequal probability of selection.

^b^ MPI score ≥ 0.33 is considered at risk of acute poverty.

^c^ P-value calculated using the independent samples t-test, adjusted for unequal probability of selection.

For younger children (6–11 months), about 20% of the RNIs of vitamin A were additionally coming from vegetable oil, while for the children aged 12–23 months, this was about one-third. In this oldest group, children from non-poor household showed a significantly higher vitamin A intake than their peers from poor households. For WRA, 37% of the RNI could be additionally covered by the fortified vegetable oil.

With regard to wheat flour, the additional iron intake is almost negligible in children 6–11 months, but for children aged 12–23 months and WRA, it adds 14% and 18% of the RNI, respectively.

**Fig 2:**
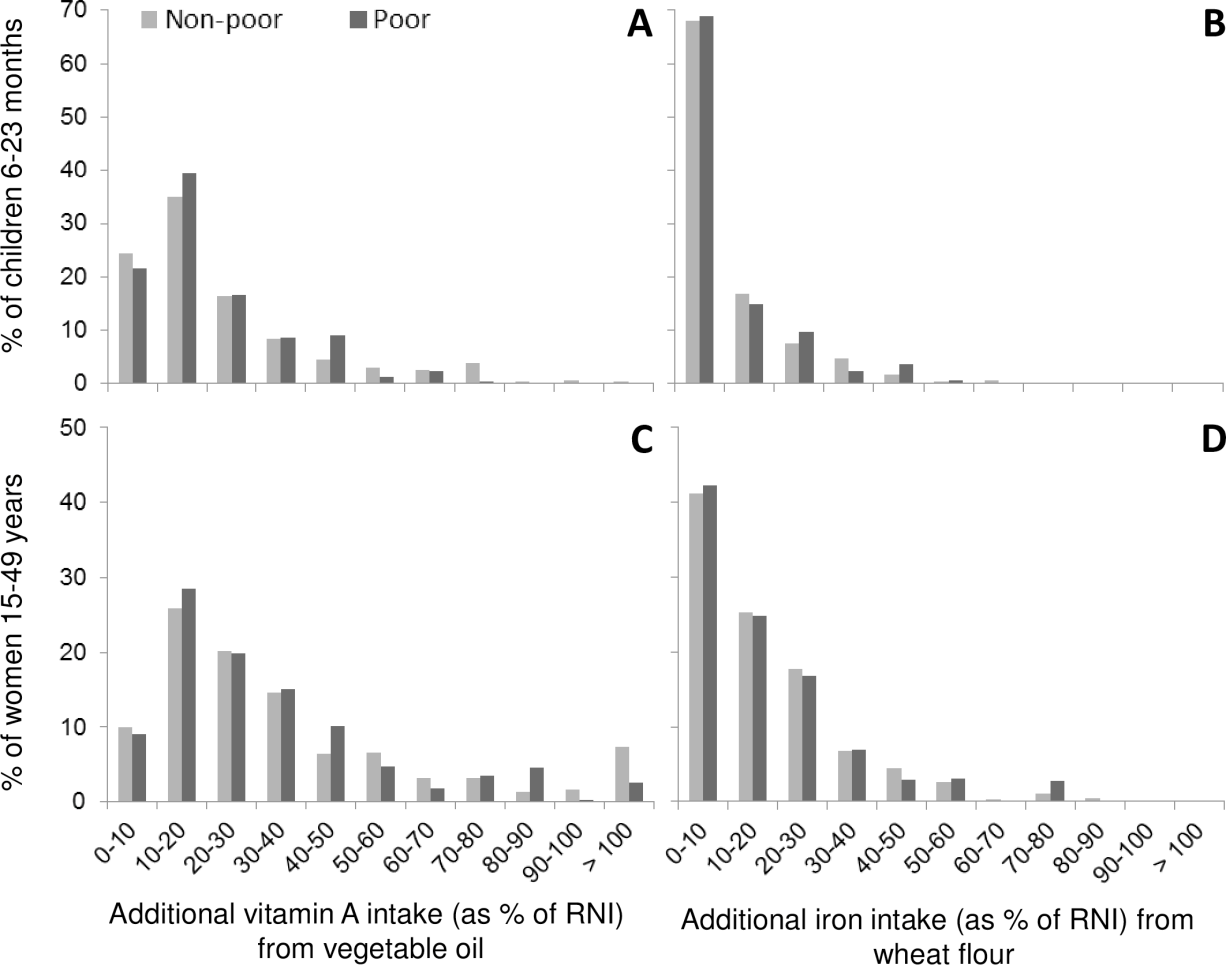
Additional Vitamin A and Iron Intake from Fortified Vegetable Oil and Wheat Flour for Children 6–23 Months of Age and Women of Reproductive Age, Disaggregated for Poor and Non-Poor Respondents, Abidjan, 2014.

As previously mentioned, Table 3 indicates that the additional micronutrient intakes between the respondents from poor and non-poor households are mostly not significantly different. In Fig 2, the additional intake of vitamin A and iron, expressed as a percent of the age-specific RNI is shown. It illustrates that while for vitamin A from vegetable oil a considerable proportion of the respondents have a considerable additional vitamin A intake (Fig 2A and 2C), for almost 70% of the children, the additional iron intake from the wheat flour is less than 10% of the RNI (Fig 2B). A similar, albeit less-pronounced, pattern can be observed for WRA in that for about two-thirds of the WRA, the additional iron intake from wheat flour is less than 20% of the RNI.

The consumption estimates of bouillon cubes revealed no statistically significant intakes between poor and non-poor WRA and children. Overall, 97% of respondents reported using bouillon cubes, with an estimated mean intake of bouillon cubes in children 6–23 months of 1.4 g/day (95% CI: 0.14, 0.15). For WRA, the estimate is 3.7 g/day (3.5, 3.9) of bouillon cubes consumed.

## Discussion

Results from the present research show that coverage with adequately fortified salt (iodine) and vegetable oil (vitamin A) is almost universal (> 85% of all samples collected) in Abidjan, the largest urban community of Côte d’Ivoire. In contrast, only about a third of the wheat flour collected showed adequate levels of iron, although all flour samples contained some additional iron but at insufficient levels. For vegetable oil, this presents an important improvement in the coverage compared to the results from the 2009 coverage survey [9], despite a profound post-electoral crisis negatively affecting food security and dietary habits [24, 25]. For wheat flour, the situation has remained suboptimal in that the majority of wheat flour does not contain adequate iron levels. Progress of adequately iodized salt is difficult to track due to a lack of previous data on adequacy. That said, previous DHS and Multiple Indicator Cluster Surveys have collected information on iodized salt (qualitatively measured using rapid test kits): In 2012, household coverage with iodized salt in Abidjan was 97% [2], which is comparable to the findings in this study showing that 95% of the salt samples collected had some iodine, albeit not all at adequate levels.

Regarding the quantities of refined vegetable oil consumption, this survey found smaller consumption figures than previously reported: For WRA living in urban areas, the mean daily oil consumption was estimated to be 43 g/day in 2009 [9] compared to just over 30 g/day in this survey. For children, the data cannot be compared, because the 2009 survey included children 6–59 months, but as the individual consumption is derived from AMEs, it is safe to assume that estimated oil consumption in children 6–23 was also higher in 2009. Using the consumption estimates and adding fortification levels into the equation, the results of this study show that about half of WRA cover 30% or more of the RNI for vitamin A, which is an important contribution to vitamin A intakes. And even for children, albeit less marked, the proportion of those with an additional vitamin A intake of more than 20% of the RNI is about half of this population group. If one takes into consideration the additional vitamin A being consumed by younger children through breast milk, vitamin A-fortified oil has the potential to complement vitamin A intake in both population groups.

Consumption estimates of wheat flour are considerably higher in the present survey than in a previous assessment: In 2006, daily wheat flour consumption among WRA living in urban areas was estimated at 84 g/day [26], compared to 125 g/day estimated in this survey, over and above the changing dietary habits since 1980 that increasingly favor consumption of processed food, including bread [27]. Furthermore, methodological differences could also have contributed to the differences: The current survey took into consideration that although a big proportion of the population consumes bread, a wide range of other wheat flour-containing products contribute to overall consumption. Although the 2006 study collected data on a few other flour-containing products, the current survey was as inclusive as possible of other wheat flour products. Another difference is that this survey was comprised only of Abidjan as an urban area, where consumption of processed food is likely higher than other urban areas. Based on results from the present survey, the current mandatory levels of 30 ppm of iron as ferrous fumarate are not in line with the WHO recommendations [28] that recommend a fortification level of 60 ppm (as ferrous fumarate) for consumption estimates of 75–149 g/day. This concern of low mandated levels of added iron in combination with only partial compliance of the actual flour samples is corroborated when looking at the additional iron intake as expressed as % RNI: More than two-thirds of the children get only negligible amounts of additional iron through fortified wheat flour (< 10% of RNI); for WRA, two-thirds get less than 20% of the RNI. If all wheat flour sampled in this survey was fortified at 60 ppm, the additional iron intake expressed as %RNI would be considerably higher: 7, 13, and 34% for children 6–8 months, 9–11 months and 12–23 months, respectively. For WRA, the additional %RNI would be around 43%. However, prior to embarking on an exercise revising mandated levels, data representing the country as a whole should be gathered to ensure that flour/flour product consumption is not higher in other parts of the country.

Although no micronutrient content analysis for bouillon cubes was performed, the consumption estimates reveal that in urban Abidjan, the bouillon cube intake is 3.7 g among WRA and 1.4 g for children aged 6–23 months old. These estimates are within the realm of previous estimations in urban areas of neighboring countries [29].

With regard to equity, the survey found that women and children from poor and non-poor households did not show large disparities in both consumption of the vehicles and fortification levels of the vehicles. Therefore, additional intakes of vitamin A and iron are comparable between the two wealth universes, although children and women from non-poor households tended to benefit slightly more. It is important to note here that the external validity of this survey is limited to Abidjan and, thus, no inferences to other parts of the country can be made.

Based on the most recent DHS survey results, less than 2% of the urban population was categorized into the two lowest wealth quintiles, while this was the case for 71% of the rural population [2]. This means that not only the proportion of households categorized as poor would be much larger if this was a national survey, but also that the level of poverty would be more pronounced. Therefore, the fact that in this survey no poor vs. non-poor differences were found does not imply that this would be the case in other urban centers or rural areas. That said, according to the most recent national census, more than one in five Ivorians live in Abidjan [30]. Nonetheless, expanding such intake estimates to the national level may be important to ensure capturing the population as a whole.

In conclusion, this survey shows that in Abidjan coverage of adequately fortified salt, wheat flour, and vegetable oil remained stable or improved since the pre-conflict period. For vegetable oil, the additional supply of vitamin A is meaningful to this urban population, and this is likely also the case for iodized salt, although no intake data were gathered. In contrast, the legally mandated iron levels in flour seem to be low compared to international recommendations and this, in combination with a small proportion of adequately fortified flour, renders the additional iron intake almost negligible, in particular among young children. Based on these findings, the national authorities should revisit the legal requirements that were recently modified to require ferrous fumarate instead of electrolytic iron as the fortificant (an improvement of the law), but simultaneously to require only 30 ppm of iron instead of the previous 60 ppm [17, 18]; such changes in legal standards should be preceded by nationally representative data to ensure appropriate modifications. Further, in view of the small proportion of flour samples compliant with the law, regulatory monitoring at production and distribution should be strengthened. Lastly, additional vehicles for iron fortification should be explored, for example, bouillon cube consumption, which was included in this survey to start this exploration.

## Supporting Information

S1 File Data statement. (DOCX)

S2 File STROBE Checklist. (DOCX)
