# Supplementary material for: The Potential of Food Fortification to Add Micronutrients in Young Children and Women of Reproductive Age – Findings from a Cross-Sectional Survey in Abidjan, Côte d’Ivoire
Source: PLoS One. 2016 Jul 6;11(7):e0158552. doi: 10.1371/journal.pone.0158552 (PMC4934880; doi:10.1371/journal.pone.0158552)
Supplement: S1 File — (DOCX) [file pone.0158552.s001.docx]

**Data Availability Statement:** Data are available by request to the Global Alliance for Improved Nutrition (GAIN). Requests can be directed to [datasharing@gainhealth.org](mailto:datasharing@gainhealth.org)

**Funding:** this work was funded by the Bill & Melinda Gates Foundation (BMGF)

**Competing interests:** All co-authors have full freedom regarding the publication of results from this study. There are no financial conflicts of interest to declare. FR, AN and JPW are employed by GroundWork LLC, a public health consultancy firm. These affiliations do not alter our adherence to all the PLoS ONE policies on sharing data and materials.

**Abbreviations:** AME, Adult Male Equivalent; GAIN, Global Alliance for Improved Nutrition; HKI, Helen Keller International; MPI, Multi-dimensional Poverty Index; PIPAF, Programme Ivoirien de la Promotion des Aliments Fortifiés (Ivorian program for the promotion of fortified foods); PSC, Pre-school age children; PSU, primary sampling unit; RNI, recommended nutrient intake; VAD, Vitamin A deficiency; WHO, World Health Organization; WRA, Women of reproductive age (15-49 y).
